# Revisiting Heterochromatin in Embryonic Stem Cells

**DOI:** 10.1371/journal.pgen.1002093

**Published:** 2011-06-02

**Authors:** Irina Stancheva

**Affiliations:** Wellcome Trust Centre for Cell Biology, University of Edinburgh, Edinburgh, United Kingdom; Netherlands Cancer Institute, The Netherlands

It is widely believed that chromatin in embryonic stem (ES) cells exists in a unique “open” conformation, characterized by sparse, disorganized heterochromatin and prevalent global transcription. Upon differentiation, this “blueprint” of pluripotent state is thought to undergo dramatic remodelling. In this issue of *PLoS Genetics*, Lienert and colleagues [1] revisit heterochromatin and transcription in pluripotent and terminally differentiated cells to demonstrate that neither the abundance of repressive histone H3 lysine 9 dimethylation (H3K9me2) nor the net transcriptional output of the genome discriminate these two very different cell states.

Pluripotent ES cells, derived from the inner cell mass of developing mammalian blastocyst, have the distinctive ability to self-renew in culture and differentiate into multiple lineages when exposed to appropriate signals. The self-organizing regulatory network of transcription factors and the epigenetic mechanisms that are involved in maintenance of pluripotent state and self-renewal are actively debated and intensively studied by many laboratories [2], [3]. When induced to differentiate, ES cells respond by changes in gene expression, cell morphology, and chromatin structure, which may collectively contribute to a reduction in developmental plasticity [4], [5].

Several lines of evidence have suggested that DNA in stem cells is packaged into an unusually dynamic form of chromatin that carries ES cell–specific patterns of histone modifications. Thus, in ES cells, histone H3 and H4 tend to be hyperacetylated; constitutive heterochromatin foci, marked by histone H3 lysine 9 trimethylation (H3K9me3), are fewer and less well organized; and histone and non-histone chromatin-bound proteins, such as heterochromatin protein 1 (HP1), are more mobile [4], [6], [7]. In addition, a substantial number of gene promoters in ES cells is marked by closely juxtaposed active (H3K4me3) and repressive (H3K27me3) chromatin modifications [8], [9]. This so-called bivalent or poised chromatin is resolved into a monovalent state at most, but not all, loci upon differentiation [9], [10]. However, repressive chromatin marks come in several “flavours”. Of those, H3K9me2 is a relatively abundant modification associated with facultative heterochromatin that covers large, gene-poor regions of the genome [11]. It has been reported that these H9K9me2 domains are “minimally present” in ES cells, but undergo substantial expansion and stabilization in differentiated tissues, such as liver and brain, resulting in transcriptional silencing of genes residing in these domains [11], [12]. Further studies have found that chromatin regions marked by other repressive modifications, such as H3K9me3 and H3K27me3, are also larger in lineage-restricted human lung fibroblasts IMR90 when compared to human ES cells. These regions undergo remodelling and reduction in size upon reprogramming of IMR90 cells into induced pluripotent stem cells (iPSCs) [10]. Collectively, these observations suggest that lineage commitment and differentiation are accompanied by expansion and stabilization of repressive chromatin.

**Figure 1:**
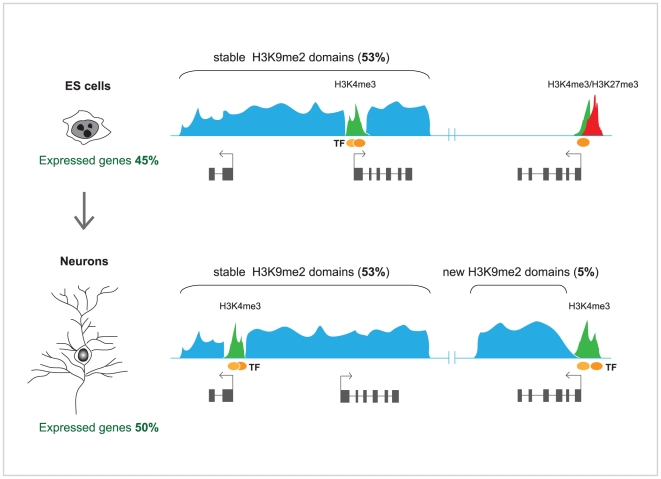
Chromatin landscapes in ES cells and terminally differentiated neurons. In ES cells, facultative heterochromatin domains marked by H3K9me2 (blue) cover a large proportion of the genome (∼53%). Terminal differentiation of ES cells into pyramidal neurons in vitro is accompanied by net gain of H3K9me2 (∼5%), mostly at new domains over the bodies of actively transcribed genes, and localized loss of H3K9me2 from much smaller regions. The focal loss of H3K9me2 could be induced by binding of specific transcription factors and modifiers (yellow/orange circles) to gene regulatory regions. Importantly, the overall size and distribution of stable H3K9me2 domains remain largely unchanged. Promoters carrying bivalent (active H3K4 [green] and repressive H3K27 [red]) marks are resolved into monovalent state during differentiation. Although different and very specific sets of genes are expressed in ES cells and neurons, the overall global transcriptional output of the genome is conserved.

In order to investigate in detail the changes in H3K9me2-marked heterochromatin domains during terminal differentiation, Lienert et al. [1] used a robust in vitro neurogenesis system to differentiate ES cells into postmitotic pyramidal neurons [13]. Profiles of H3K9me2, representing ∼10% of the genome, including the entire chromosome 19, were generated for both cell types and compared to each other. Surprisingly, it was found that these profiles showed high degree of correlation between ES cells and neurons. In both cell types, H3K9me2 covered ∼50% of chromosome 19, and a very modest increase in H3K9me2 (5%) was observed in terminally differentiated neurons. In agreement with an earlier study [11], H3K9me2 was enriched at large chromosomal domains, but those were generally invariable in median size and distribution between ES cells and neurons, and mutually exclusive with active (H3K4me2) and other repressive chromatin marks (H3K27me3). Some discrete differences were observed; those included gain of H3K9me2 over new large domains in neurons, mostly over the bodies of transcribed genes, as well as loss of H3K9me2 from much smaller regions (Figure 1). Furthermore, high throughput sequencing of RNA (RNA-seq) from ES cells, neurons, and, additionally, mouse embryonic fibroblasts, showed well defined cell type–specific expression, but no significant overall difference in the transcribed portion of the genome, including most repetitive sequences. Although the findings of Lienert et al. [1] seem to disagree with previous studies [4], [11], these discrepancies could be largely explained by methodological differences in the analyses of H3K9me2 genomic microarray data [12] and the accuracy in discriminating between low and absent transcription by microarrays, which may suffer from crosshybridization, versus unambiguous direct counting of RNA sequence reads [1]. As both Effroni et al. [4] and Lienert et al. [1] have measured the abundance of polyadenylated RNAs, reflecting mostly the productive transcription, it might be interesting to employ global nuclear run-on coupled with high throughput sequencing (NRO-seq) [14] in order to explore whether the extent of non-productive transcription differs significantly between ES cells and terminally differentiated neurons.

In summary, the observations of Lienert et al. [1] highlight the remarkable conservation of the facultative heterochromatin domains and the global transcriptional output of the genome between ES cells and terminally differentiated neurons. They also suggest that genome reprogramming during lineage commitment and differentiation is largely achieved by developmental cues and strong transcription factors, which induce localized and highly specific changes in heterochromatin rather than promote genome-wide build up of H3K9me2 and suppression of global low-level transcription. Such a model is further supported by findings that differentiation of ES cells into neuronal progenitors and then into astrocytes is accompanied by focal, localized rearrangements in chromatin-nuclear lamina interactions, while the overall architecture of lamina-associated chromosomal domains remains largely preserved [15].

It cannot be completely ruled out that, although quantitatively similar, heterochromatin is qualitatively different, more fluid and, perhaps, less essential in ES cells than in terminally differentiated cells and tissues. Such plasticity could be mediated by chromatin remodelling ATPases, histone acetyltransferases, and histone demethylases, some of which are highly expressed in stem cells and essential for pluripotency [4], [16]–[18]. Is heterochromatin then functional in ES cells?

The vast majority of H3K9me2 in the genome is established by the euchromatic histone methylases EHMT2 and EHMT1, also known as G9a and GLP, respectively. Similar to the knockouts of DNA methyltransferases [19], ES cells lacking either G9a or GLP are viable and morphologically normal, but *G9a^−/−^* and *Glp^−/−^* embryos die in midgestation (E9–9.5) [20], [21]. This suggests that, although DNA methylation and G9a/GLP-dependent H3K9me2 are dispensable for self-renewal in ES cells, they become vital during differentiation and embryonic development. Unfortunately, the differentiation potential of *G9a^−/−^* and *Glp^−/−^* ES cells has never been investigated in detail. Nevertheless, these cells form embryonic bodies upon induction with retinoic acid, but fail to terminally silence OCT3/4 [22], indicating that G9a/GLP-dependent heterochromatin formation may safeguard rather than actively channel differentiation.

Despite the overwhelming evidence that heterochromatin is present, but somewhat “wimpy” in stem cells, it was reported that H3K9me2- and H3K9me3-specific histone demethylases JMJD1A and JMJD2C, respectively, are directly regulated by OCT3/4 transcription factor and are essential for maintenance of pluripotency [18]. Depletion of these enzymes by small interfering RNAs (siRNAs) leads to accumulation of H3K9me and unscheduled differentiation. However, it was also clearly shown that JMJD1A and JMJD2C action is restricted to specific loci and does not lead to ubiquitous removal of H3K9me from the genome. Taken together with the studies of Lienert et al. [1], these findings firmly indicate that heterochromatin is functional in ES cells and has to be actively remodelled in order to allow the self-organizing network of transcription factors to prevent differentiation and promote self-renewal. The same general principle of local heterochromatin removal by lineage-specific transcriptional regulators may operate during differentiation.
